# Angiography-based hemodynamic features predict recurrent ischemic events after angioplasty and stenting of intracranial vertebrobasilar atherosclerotic stenosis

**DOI:** 10.1007/s00330-023-10209-x

**Published:** 2023-09-19

**Authors:** Kangmo Huang, Weihe Yao, Mingming Zha, Shanmei Qin, Yingle Li, Yan Xu, Rui Liu, Ruidong Ye, Yunfei Han, Wusheng Zhu, Zhongzhao Teng, Juan Du, Xinfeng Liu

**Affiliations:** 1 Department of Neurology, Affiliated Jinling Hospital, Medical School of Nanjing University, Nanjing, China; 2 Department of Neurology, Jinling Hospital, The First School of Clinical Medicine, Southern Medical University, Nanjing, China; 3 Neusoft Medical Systems Co., Ltd., Shenyang, China; 4 Department of Radiology, University of Cambridge, Cambridge, UK

**Keywords:** Hemodynamics, Intracranial atherosclerosis, Cerebral angiography, Recurrence, Stroke

## Abstract

**Objectives:**

To assess the predictive value of hemodynamic features for stroke relapse in patients with intracranial vertebrobasilar atherosclerotic stenosis treated with percutaneous transluminal angioplasty and stenting (PTAS) using quantitative digital subtraction angiography (q-DSA).

**Methods:**

In this retrospective longitudinal study, patients with intracranial vertebrobasilar atherosclerotic stenosis and who underwent PTAS treatment between January 2012 and May 2020 were enrolled. The q-DSA assessment was performed before and after PTAS. ROIs 1–4 were placed along the vertebral artery, proximal and distal basilar artery, and posterior cerebral artery; ROIs 5–8 were in 5 mm and 10 mm proximal and distal to the lesion, respectively. Relative time to peak (rTTP) was defined as the difference in TTP between ROIs. Cox regression analyses were performed to determine risk factors for recurrent stroke.

**Results:**

A total of 137 patients (mean age, 62 years ± 10 [standard deviation], 83.2% males) were included, and 26 (19.0%) patients had stroke relapse during follow-up (median time of 42.6 months [interquartile range, 19.7–60.7]). Preprocedural rTTP_4-1_ (adjusted hazard ratio (HR) = 2.270; 95% CI 1.371–3.758; *p *= 0.001) and preprocedural rTTP_8-5_ (adjusted HR = 0.240; 95% CI 0.088–0.658; *p *= 0.006) were independently associated with the recurrent stroke. These hemodynamic parameters provided an incremental prognostic value for stroke relapse (AUC, 0.817 [0.704–0.931]; the net reclassification index, 0.431 [0.057–0.625]; and the integrated discrimination index, 0.140 [0.035–0.292]).

**Conclusions:**

In patients with intracranial vertebrobasilar atherosclerosis treated with PTAS, preprocedural prolonged TTP of the target vessel and shortened trans-stenotic TTP difference were associated with stroke relapse. Q-DSA-defined hemodynamic parameters provided incremental predictive value over conventional parameters for stroke recurrence.

**Clinical relevance statement:**

Quantitative DSA analysis enables intuitive observation and semi-quantitative evaluation of peri-therapeutic cerebral blood flow. More importantly, quantitative DSA–defined hemodynamic parameters have the potential for risk stratification of patients with intracranial atherosclerotic stenosis.

**Key Points:**

- *Semi-quantitative angiography-based parameters can reflect pre- and postprocedural subtle changes in blood flow in patients with intracranial atherosclerotic stenosis.*
- *Although angioplasty procedures can significantly improve blood flow status, patients with more restricted baseline blood flow still show a higher risk of stroke recurrence.*
- *Angiography-based hemodynamic features possess prognostic value and can serve as clinical markers to assess stroke risk of patients with intracranial atherosclerotic stenosis.*

**Supplementary Information:**

The online version contains supplementary material available at 10.1007/s00330-023-10209-x.

## Introduction

Intracranial atherosclerotic stenosis (ICAS) is a major cause of ischemic stroke worldwide [1], accounting for almost 30% of ischemic strokes in Asia [2]. Hypoperfusion and artery-to-artery embolism caused by ICAS are important mechanisms leading to ischemic stroke [3]. Patients with symptomatic ICAS have a stroke risk of over 10% per year, despite optimal medical treatment [4, 5]. Although not widely applied to the general ICAS population, percutaneous transluminal angioplasty and stenting (PTAS) and angioplasty alone are still important treatment options for high-risk or relapsed ICAS after ineffective medical treatment. Moreover, PTAS undergoing balloon angioplasty before or after stent implantation could achieve better stenosis relief and perfusion improvement. Earlier pivotal trials indicated an apparent increase in stroke and death in patients with ICAS after stenting [5, 6]. In contrast, recent studies revealed positive results of PTAS for symptomatic ICAS with experienced interventionalists and proper patient selection [7, 8]. Current data show that the 1-year recurrence rate of ICAS patients treated with PTAS is around 8.0–8.5%.

To improve the benefit of PTAS treatment, it is crucial to perform comprehensive assessments and identify the risk factors of long-term recurrent stroke. Compared with luminal stenosis, pilot studies have shown that detailed intracranial atherosclerotic features and hemodynamic status were more relevant to clinical presentations and subsequent ischemic events [9–12]. Digital subtraction angiography (DSA)–defined parameters, in particular, those before and after stent deployment, observed or measured during the PTAS have not been paid sufficient attention. DSA is the current gold standard for luminal stenosis measurement. It, however, can also provide semi-quantitative parameters as a supplement for classical hemodynamic values. Although this can also be quantified by computed tomography perfusion, magnetic resonance perfusion–weighted imaging, and arterial spin labeling, the artifacts of posterior cranial fossa and anatomical vascular variations may hinder the detailed evaluation of the hemodynamic of ICAS in the posterior circulation. Quantitative DSA (q-DSA) allows a real-time evaluation of hemodynamics with superior temporal and spatial resolution. Its feasibility for evaluating blood flow changes has been demonstrated in various cerebrovascular diseases, such as cerebral vasospasm, arteriovenous malformations, and steno-occlusive arterial disease [13–17].

Quantitative DSA can detect subtle changes in blood flow in the angiography suite during the intervention [18]. The main aim of this study is, therefore, to identify the clinically relevant pre- and postprocedural hemodynamic parameters derived from q-DSA, and assess their potential prognostic values in patients with posterior ICAS who underwent PTAS therapy.

## Materials and methods

### Data availability statement

All relevant raw data will be made available by the corresponding author upon request.

### Patients

This retrospective study was approved by the Internal Review Board of local hospital and was performed in accordance with the ethical standards as laid down in the 1964 Declaration of Helsinki and its later amendments or comparable ethical standards. The requirement for informed consent was waived. In addition, the study was registered at the Chinese Clinical Trial Registry with ID: ChiCTR2100053823 (Registration URL: https://www.chictr.org.cn).

From January 2012 to May 2020, the database of the prospective Stroke Registry Program [19] was retrospectively reviewed for patients with ischemic stroke or transient ischemic attack (TIA). Inclusion criteria were as follows: (a) age >18 years, (b) ICAS with luminal stenosis ≥ 50% and < 100% (nonoccluded lesion), (c) the stenotic lesion in the posterior circulation (the intradural vertebral artery [VA4] or basilar artery), and (d) receiving PTAS procedures after hyperacute phase (more than 48 h after onset). Exclusion criteria included the following: (a) patients with disturbance of consciousness, (b) failure of device placement, (c) missing DSA image or poor image quality, (d) loss of follow-up.

Baseline clinical characteristics including demographics (age, gender, blood pressure, and body mass index), medical histories (hypertension, diabetes, coronary heart disease, prior stroke, TIA, and smoke), the baseline National Institutes of Health Stroke Scale score, the baseline modified Rankin Scale score, and laboratory tests after admission were collected from the databases. Patients were interviewed either at the outpatient or via telephone on the 3rd, 6th, and 12th months after the procedure and annually thereafter. The primary endpoint was stroke relapse occurring with new brain infarction confirmed by imaging. For suspected stroke recurrence without radiographic confirmation, a neurologist would make the final diagnosis through the clinical features and duration. The secondary endpoints were recurrent ischemic events, including recurrent stroke and TIA.

### Image acquisition and analysis

All angiograms were performed with a standard, routine clinical protocol using a biplane flat-panel system (Artis zee, Siemens Healthcare GmbH). A heparinized saline-flushed 6-French or 8-French guide catheter was placed proximally in vascular lesions for diagnostic angiography. The contrast agent iodixanol (320 mg/mL, Visipaque, GE Healthcare) was injected into the intracranial artery by a power injector (Medrad, Bayer HealthCare) at a flow rate of 4 mL/s for 1.5 s. The power injector was synchronized to the angiographic system with a 1.0-s delay after injection. DSA images were acquired at a rate of 4 frame/s.

Luminal stenosis was measured in DSA following the Warfarin-Aspirin Symptomatic Intracranial Disease (WASID) method [20] (= [1 − diameter of stenotic artery/diameter of proximal normal artery] × 100%). The angiographic characteristics of ICAS lesions were classified according to the Mori lesion classification, and were briefly summarized as follows: type A, short (≤ 5 mm in length) concentric or moderately eccentric; type B, tubular (5 to 10 mm in length) or extremely eccentric; type C, diffuse (> 10 mm in length) or extremely angulated (> 90°) [21]. The hemodynamics analyses were performed offline in a new prototype software, Atlas (Neusoft Medical Systems Co., Ltd.) The time-varying contrast intensity of each pixel throughout the angiogram cycle was identified to generate the time-density curve and calculate parametric color-coded DSA maps [22, 23].

**Fig. 1:**
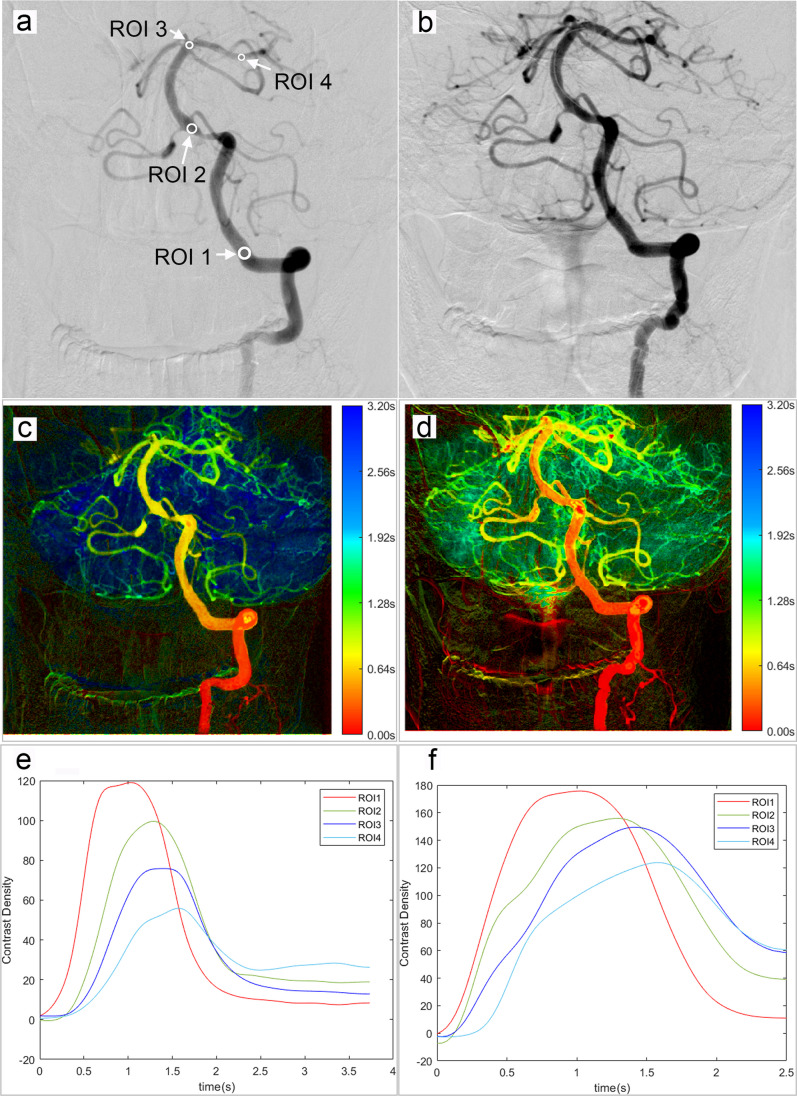
Pre- and postprocedural hemodynamic analyses by quantitative digital subtraction angiography. These show the original DSA images (**a**, **b**), quantitative DSA images which were color-coded according to the values of time to peak (**c**, **d**), and the time-density curve of selected ROIs (**e**, **f**) before (**a**, **c, e**) and after (**b**, **d**, **f**) the procedure. In this case, it was observed that the stenosis of the fusion segment of the basilar artery was relieved and the cerebral blood perfusion status was significantly improved after the procedure. Abbreviations: ROI, regions of interest

For the target vessel, four ROIs were placed in the intradural vertebral artery (VA4) and the proximal and distal segment of the basilar artery, as well as the junction of the posterior cerebral artery first segment (P1) and the second segment (P2), as shown in Fig. 1. ROIs were successively marked as ROI 1, ROI 2, ROI 3, and ROI 4 from the proximal towards the distal vessel. For the target lesion, ROI 5 and ROI 6 were placed at 5 mm and 10 mm proximal to the target lesion, and ROI 7 and ROI 8 were placed at 5 mm and 10 mm distal to the target lesion, respectively. In addition, the ROIs were set on identical locations of similar viewing projections in the pre- and postoperative DSA series. The diameter of each ROI was the same as the local vessel diameter.

The time-density curve of each ROI was obtained to elucidate hemodynamic parameters. Time to peak (TTP) was defined as the time at the maximum contrast concentration. Relative TTP (rTTP) was the difference between the TTP of distal ROIs and those at the proximal (e.g., rTTP_4-1 _= TTP_ROI4_ − TTP_ROI1_) to minimize the possible impact of the catheter positioning and injection protocol. The AUC value was the area of the time-density curve for each ROI, which indirectly reflected the blood volume within the ROI. The relative AUC (rAUC) was evaluated as the ratio of distal and proximal ROIs (e.g., rAUC_4/1 _= AUC_ROI4_/AUC_ROI1_). Further normalization was achieved by calculating the relative values of pre- and postprocedural parameters (e.g., Δ rTTP_4-1 _= Preprocedural rTTP_4-1_ − Postprocedural rTTP_4-1_; relative rAUC_4/1 _= Preprocedural rTTP_4-1_/Postprocedural rTTP_4-1_).

### Statistical analyses

Data were described as medians (interquartile range, IQR) or means (standard deviation, SD) for continuous variables depending on their distribution, and numbers (percentage) for categorical variables. The differences between patients with or without recurrent events were compared using the Student *t*-test, Mann-Whitney *U*, chi-square, or Fisher exact test, where appropriate; otherwise, a Wilcoxon signed-ranks test was used for pairwise comparisons. X-tile software was used to determine the optimal cutoff value.

Confounding factors and variables with a value of *p* < 0.1 in the univariate Cox regression were selected for multivariable Cox models with a forward stepwise selection. Model-1 was based on the clinical variables, without considering any hemodynamic parameters; model-2 was based on the hemodynamic parameters only; and model-3 included both clinical variables and hemodynamic parameters. For each model, the ROC curve was generated to calculate the area under the ROC curve (AUC). Net reclassification index and integrated discrimination index were determined to assess the incremental value of hemodynamic parameters over the traditional risk factors, that is, to assess the difference between model-3 and model-1. Kaplan-Meier curves were plotted to estimate the cumulative probabilities of recurrent ischemic events by dichotomized rTTP.

Subgroup analyses were performed to assess the interactions between dichotomized rTTP and certain patient characteristics, in predicting recurrent stroke. Two-sided *p* < 0.050 were considered to be statistically significant. Statistical analyses were performed with SPSS (version 26, IBM-Armonk) and R statistical software (version 4.0.3).

## Results

From January 2012 to May 2020, 307 ICAS patients who underwent endovascular therapy were registered in the Nanjing Stroke Registry Program, and a total of 137 patients were included in this study. The flow chart of patient selection is summarized in Supplementary Fig. 1. Patients were 62.1 ± 9.7 years, and 114 (83.2%) were men. Of these 137 patients, 129 patients (94.2%) had ischemic strokes and 8 TIA. Ninety-eight (71.5%) lesions located in vertebral artery (VA) and 39 (28.5%) in basilar artery (Table 1). One hundred and eight cases were identified as Mori A or B type, and 29 cases were Mori C type. The detailed characteristics of all patients are listed in Table 1.

**Table 1:** Clinical characteristics of patients with intracranial atherosclerotic stenosis who underwent percutaneous transluminal angioplasty and stenting

	Overall	Without recurrence	With recurrence	*p* value
	(*n *= 137)	(*n *= 111)	(*n *= 26)	
Demographics				
Age, mean (SD)	62.1 (9.7)	62.3 (9.4)	61.0 (11.1)	0.660
Male, *n* (%)	114 (83.2)	93 (83.8)	21 (80.8)	0.772
SBP, median (IQR)	138 (130–150)	138 (130–150)	138 (130–150)	0.792
BMI, median (IQR)	25.4 (23.9–26.9)	25.4 (23.9–26.8)	25.8 (24.1–27.9)	0.207
Medical history, *n* (%)				
Hypertension	115 (83.9)	93 (83.8)	22 (84.6)	1.000
Diabetes	52 (38.0)	41 (36.9)	11 (42.3)	0.611
CHD	15 (10.9)	14 (12.6)	1 (3.8)	0.302
Smoke	64 (46.7)	51 (45.9)	13 (50.0)	0.709
Prior stroke	50 (36.5)	39 (35.1)	11 (42.3)	0.494
Prior TIA	29 (21.2)	20 (18.0)	9 (34.6)	0.062
NIHSS score, median (IQR)	0 (0–4)	1 (0–4)	0 (0–3)	0.187
mRS score, median (IQR)	1 (1–2)	1 (1–2)	1 (1–2)	0.544
Laboratory findings, median (IQR)				
Glu, mmol/L	5.3 (4.7–6.7)	5.3 (4.7–6.5)	5.7 (4.7–7.1)	0.480
NLR	2.35 (1.77–3.30)	2.30 (1.76–3.20)	2.66 (1.92–4.09)	0.144
TC, mmol/L	3.81 (3.00–4.52)	3.83 (3.01–4.55)	3.46 (2.84–4.37)	0.296
TG, mmol/L	1.41 (1.01–1.94)	1.42 (0.99–1.98)	1.35 (1.08–1.79)	0.848
HDL, mmol/L	0.96 (0.84–1.16)	0.99 (0.85–1.17)	0.88 (0.85–0.98)	0.060
LDL, mmol/L	2.16 (1.64–2.71)	2.16 (1.69–2.74)	2.05 (1.20–2.51)	0.173
Qualifying event, *n* (%)				0.647
Stroke	129 (94.2)	105 (94.6)	24 (92.3)	
TIA	8 (5.8)	6 (5.4)	2 (7.7)	
Lesion localization, *n* (%)				0.440
Vertebral artery	98 (71.5)	81 (73.0)	17 (65.4)	
Basilar artery	39 (28.5)	30 (27.0)	9 (34.6)	
Mori type, *n* (%)				0.660
A type	69 (50.4)	56 (50.5)	13 (50.0)	
B type	39 (28.5)	33 (29.7)	6 (23.1)	
C type	29 (21.2)	22 (19.8)	7 (26.9)	
Interval from onset to PTAS, days, median (IQR)	57 (37–78)	58 (34–79)	52 (39–63)	0.314
Device, *n* (%)				0.941
Stent	37 (27.0)	31 (27.9)	6 (23.1)	
Balloon	12 (8.8)	10 (9.0)	2 (7.7)	
Stent + balloon	88 (64.2)	70 (63.1)	18 (69.2)	
Preprocedural stenosis degree, %, median (IQR)	76 (69–83)	76 (69–83)	75 (70–85)	0.725
Postprocedural stenosis degree, %, median (IQR)	19 (7–32)	19 (7–32)	16 (1–32)	0.307

Abbreviations: *SD*, standard deviation; *IQR*, interquartile range; *SBP*, systolic blood pressure; *BMI*, body mass index; *CHD*, coronary heart disease; *TIA*, transient ischemic attacks; *ESRS*, Essen Stroke Risk Score; *NIHSS*, National Institute of Health stroke scale; *mRS*, the modified Rankin Scale; *NLR*, neutrophil-lymphocyte ratio; *TC*, total cholesterol; *TG*, triacylglycerol; *HDL*, high-density lipoprotein; *LDL*, low-density lipoprotein; *PTA*, percutaneous transluminal angioplasty and stenting

### Factors associated with recurrent ischemic events

The median follow-up was 42.6 months (IQR, 19.7–60.7 months) for all patients. During the follow-up, 26 (19.0%) patients experience a recurrent stroke. Compared with those without recurrence, patients with recurrent events had more previous TIA before admission (34.6% vs. 18.0%, *p *= 0.062) and lower levels of high-density lipoprotein (HDL, 0.88 mmol/L vs. 0.99 mmol/L, *p *= 0.060); however, the differences were not significant. Other clinical characteristics of the two cohorts were comparable (Table 1).

**Fig. 2:**
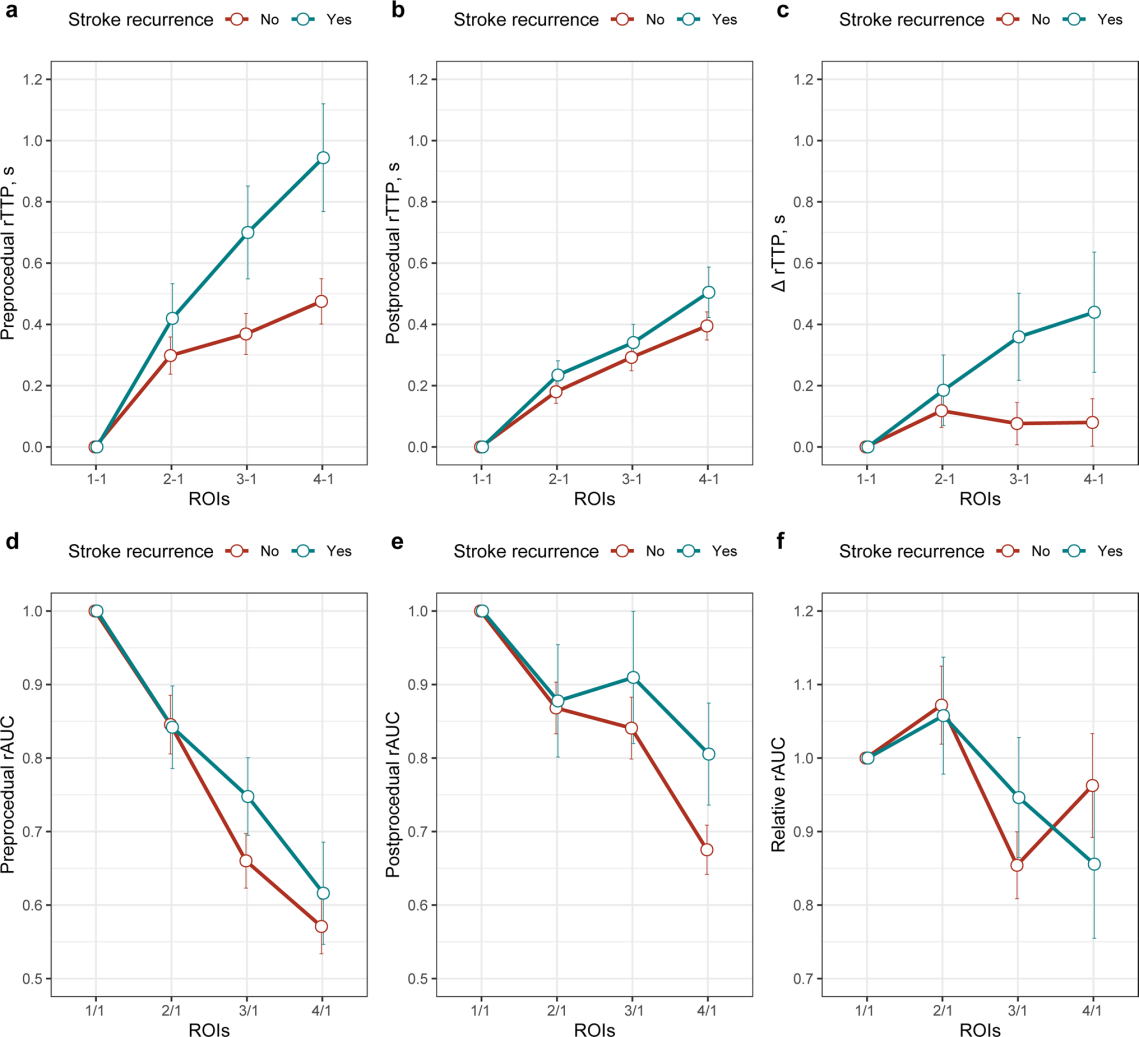
The comparison of pre- and postprocedural hemodynamic parameters at different ROIs of the target vessel between patients with and without recurrent stroke. The first row shows the values of preprocedural (**a**) and postprocedural (**b**) rTTP, and the change of rTTP (**c**), and the second row shows preprocedural (**d**) and postprocedural (**e**) rAUC, and the relative rAUC (**f**). The green lines represent parameters of patients with recurrent stroke; the red lines represent parameters of patients without recurrent stroke. Abbreviations: ROI, regions of interest; rTTP, relative time to peak; rAUC, relative area under the curve

**Fig. 3:**
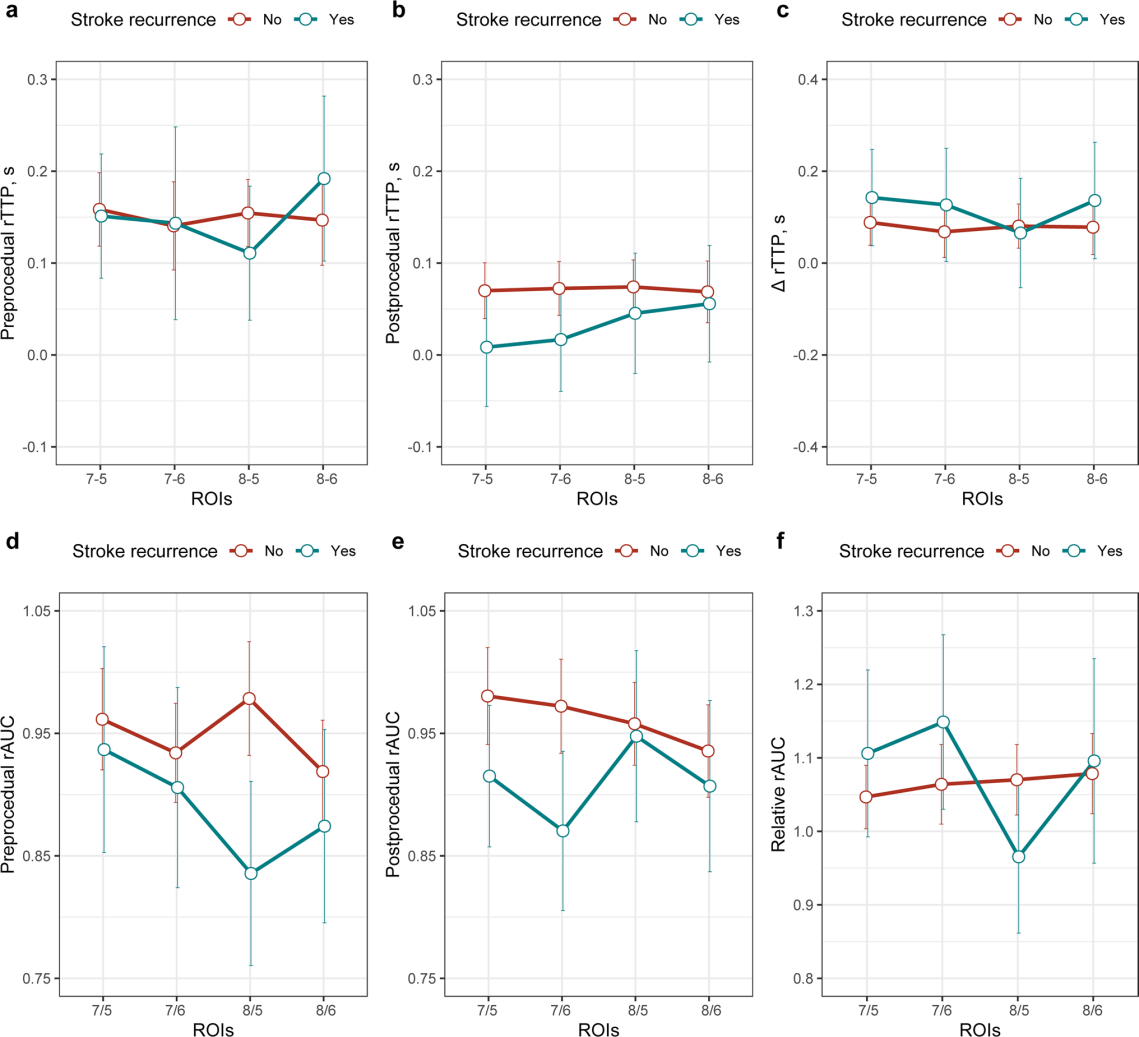
The comparison of pre- and postprocedural hemodynamic parameters at trans-stenotic ROIs between patients with and without recurrent stroke. The first row shows the values of preprocedural (**a**) and postprocedural (**b**) rTTP, and the change of rTTP (**c**), and the second row shows preprocedural (**d**) and postprocedural (**e**) rAUC, and the relative rAUC (**f**). The green lines represent parameters of patients with recurrent stroke; the red lines represent parameters of patients without recurrent stroke. Abbreviations: ROI, regions of interest; rTTP, relative time to peak; rAUC, relative area under the curve

TTP and AUC values of each ROI were derived from DSA images. Overall, compared with the preprocedural data, the postprocedural rTTPs were significantly shorter (all *p*s < 0.05), and postprocedural rAUC_3/1_ (= AUC_ROI3_/AUC_ROI1_) and rAUC_4/1_ (= AUC_ROI4_/AUC_ROI1_) were significantly increased (*p*s < 0.001). Figure 2 illustrates the pre- and postprocedural hemodynamic parameters at different ROIs of the target vessel. Compared with patients without recurrent stroke, patients who experienced recurrent stroke tended to have longer rTTPs, especially the preprocedural rTTP_4-1_ (0.64 s vs. 0.53 s, *p *= 0.035) (Fig. 2a). Figure 3 shows the pre- and postprocedural hemodynamic parameters at trans-stenotic ROIs. Patients with recurrent stroke tended to have lower rTTP_8-5_ (0.09 s vs. 0.13 s, *p *= 0.079; Fig. 3a) and lower rAUC_8/5_ (0.71 vs. 0.86, *p *= 0.159; Fig. 3d) than those free from recurrent stroke. In general, the difference in hemodynamic parameters between the two groups became smaller after the procedure (Figs. 2 and 3).

**Fig. 4:**
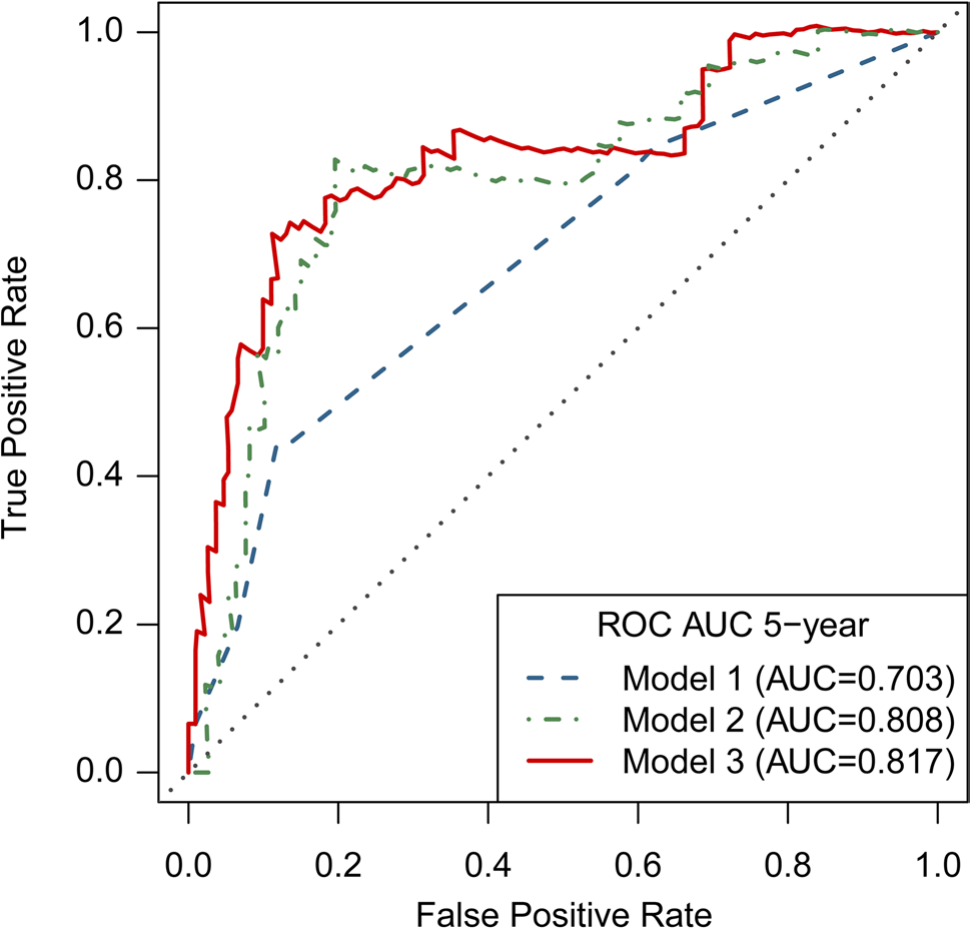
Time-dependent ROC curves (at 5 years) of prediction models. The blue dashed lines represent the ROC curve of model-1 (AUC = 0.703); the green dot-dashed lines represent the ROC curve of model-2(AUC = 0.808); the red solid lines represent the ROC curve of model-3 (AUC = 0.817)

Traditional risk factors and variables with *p* < 0.1 in the univariate analysis were selected for the multivariable analysis. As shown in Table 2, model-1 included high-risk clinical factors only that were thought to be associated with cardiovascular events: previous TIA, neutrophil-to-lymphocyte ratio (NLR, dichotomized by the optimal cutoff value [4.03]), and dichotomized HDL (< 1.04 mmol/L and ≥ 1.04 mmol/L). Model-2 included potential predictive hemodynamic parameters only with *p* < 0.1 in the univariate analysis: preprocedural rTTP_4-1_, preprocedural rTTP_8-5_ (dichotomized by the optimal cutoff value [0.14 s]), and preprocedural rAUC_8/5_ (dichotomized by the optimal cutoff value [0.89]). Model-3 was developed by pooling variables included in models 1 and 2. Results indicated that model-3 had the best prognostic performance (AUC = 0.817; 95% CI, 0.704–0.931), model-2 performed relatively well (AUC = 0.808; 95% CI, 0. 691–0.926), and model-1 was the poorest (AUC = 0.703; 95% CI, 0.571–0.835) (Fig. 4).

**Table 2:** Multivariate Cox regression models showing the predictive power of different risk factors in combination

	Model-1^a^		Model-2^b^		Model-3^c^	
	HR (95% CI)	*p* value	HR (95% CI)	*p* value	HR (95% CI)	*p* value
Prior TIA (Yes)	2.479 (1.099–5.594)	0.029			2.460 (1.062–5.699)	0.036
NLR (> 4.03)	2.953 (1.232–7.075)	0.015			3.042 (1.228–7.538)	0.016
HDL (< 1.04 mmol/L)	2.970 (1.113–7.924)	0.030			3.064 (1.126–8.333)	0.028
rTTP_4-1_			2.121 (1.321–3.405)	0.002	2.270 (1.371–3.758)	0.001
rTTP_8-5_ (> 0.14 s)			0.231 (0.083–0.642)	0.005	0.240 (0.088–0.658)	0.006
rAUC_8/5_ (> 0.89)			0.393 (0.156–0.989)	0.047		

^a^Model-1 was established by variables (age, body mass index, hypertension, diabetes, atrial fibrillation, prior TIA, smoke, blood glucose, HDL, NLR, the interval from onset to PTAS) using stepwise forward selection

^b^Model-2 was established by hemodynamic parameters (rTTP_4-1_, rTTP_3-1_, rTTP_8-5_, rTTP_8-6_, rAUC_8/5_) using stepwise forward selection

^c^Model-3 was established by variables (age, body mass index, hypertension, diabetes, atrial fibrillation, prior TIA, smoke, blood glucose, HDL, NLR, interval from onset to PTAS, rTTP_4-1_, rTTP_8-5_, rAUC_8/5_) using stepwise forward selection. Abbreviations: *HR*, hazard ratios; *CI*, confidence interval; TIA, transient ischemic attacks; *NLR*, neutrophil-lymphocyte ratio; *HDL*, high-density lipoprotein; *rTTP*, relative time to peak; *rAUC*, relative area under the curve

Compared with traditional high cardiovascular risk factors only, the combination with hemodynamic parameters improved the prognostic discriminatory power with a significant incremental improvement of the continuous net reclassification index (0.431; 95% CI, 0.057–0.625; *p *= 0.019) and the integrated discrimination index (0.140; 95% CI, 0.035–0.292; *p *= 0.004).

**Fig. 5:**
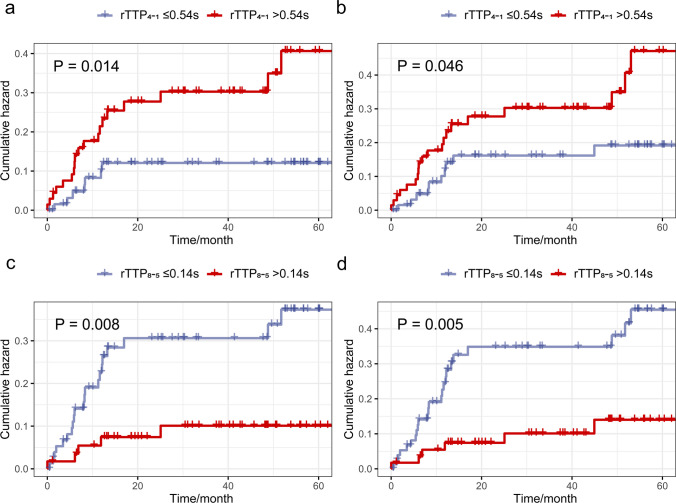
The Kaplan-Meier curves for recurrent ischemic events of cohorts with different rTTPs (relative time to peak). Kaplan-Meier curves for recurrent stroke (**a**) and recurrent ischemic events (ischemic stroke or transient ischemic attacks, panel **b**) of intracranial atherosclerotic stenosis (ICAS) patients with different rTTP_4-1_ values, and for recurrent stroke (**c**) and recurrent ischemic events (**d**) of ICAS patients with different rTTP_8-5_ values

In particular, preprocedural rTTP_4-1_ (adjusted hazard ratio (HR) = 2.270; 95% CI 1.371–3.758; *p *= 0.001) and rTTP_8-5_ (adjusted HR = 0.240; 95% CI 0.088–0.658; *p *= 0.006) were independently associated with the stroke recurrence. The Kaplan-Meier curves indicated higher preprocedural rTTP_4-1_ (> 0.54 s) was associated with a higher risk of recurrent stroke (log-rank *p *= 0.014; Fig. 5 a) and recurrent ischemic events (log-rank *p *= 0.046; Fig. 5 b), and lower preprocedural rTTP_8-5_ (≤ 0.14 s) was also significantly associated with a higher risk of recurrent stroke (log-rank *p *= 0.008; Fig. 5 c) and recurrent ischemic events (log-rank *p *= 0.005; Fig. 5 d).

### Subgroup analyses

**Fig. 6:**
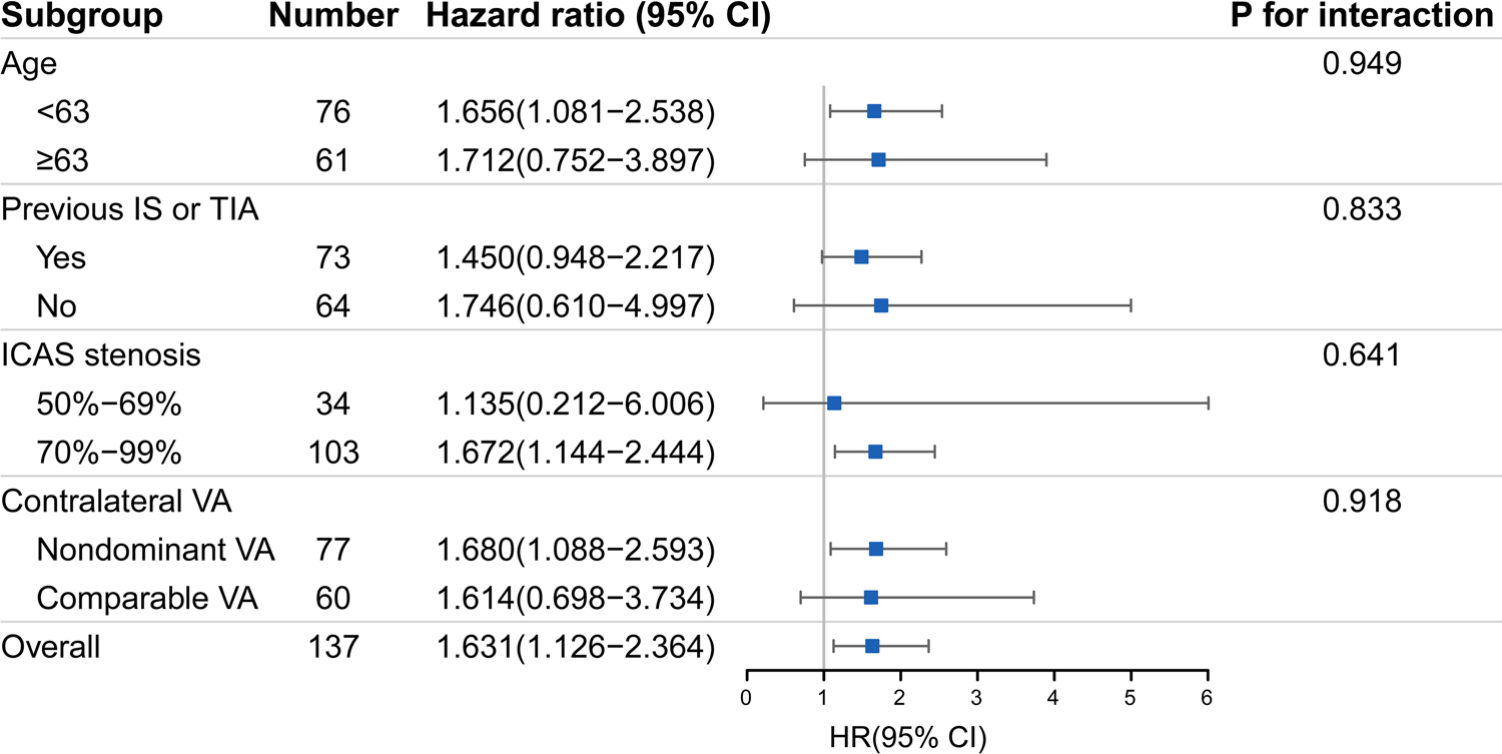
Subgroup analyses of recurrent ischemic stroke. This forest plot shows the hazard ratios and 95% confidence intervals for the relative risk between patients with high and low rTTP_4-1_ before the percutaneous transluminal angioplasty procedure. Abbreviations: rTTP, relative time to peak; IS, ischemic stroke; TIA, transient ischemic attack; ICAS, intracranial atherosclerosis; VA, vertebral artery

Subgroup analyses were stratified by age (the median age: < 63 years vs. ≥ 63 years), previous ischemic stroke or TIA (with/without), degree of ICAS stenosis (50–69% vs. 70–99%), and the status of contralateral VA (nondominant vs. comparable VA). In subgroup analyses, prolonged rTTP_4-1_ remained associated with an increased risk of recurrent stroke. No significant interactions were detected between the dichotomized rTTP_4-1_ and those characteristics in recurrent stroke (all *p* for interaction > 0.1; Fig. 6).

## Discussion

This is a longitudinal study evaluating the prognostic value of hemodynamic parameters derived from q-DSA in ICAS patients treated with PTAS. Several inspiring findings emerged from this study. Above all, even though the PTAS procedure markedly improved the blood flow of the target vessels, the preprocedural hemodynamic status was still significantly related to the prognosis. The prolonged rTTP of the target vessel and shortened translesional TTP difference derived from DSA imaging before the procedure were independently associated with an increased risk of stroke relapse. Secondly, prior TIA, low levels of HDL, and high NLR were also identified as the risk factors for subsequent ischemic stroke. Thirdly, multivariate analyses showed that hemodynamic parameters provided an incremental prognostic value over traditional risk factors for recurrent stroke prediction.

For the first time, we conducted a longitudinal study to evaluate the prognostic value of hemodynamic features in posterior ICAS patients treated with PTAS, using the q-DSA technique. Q-DSA has been used to evaluate subtle hemodynamic changes to determine the treatment efficacy for several cerebrovascular diseases, e.g., cerebral aneurysms, cerebral vasospasm, arteriovenous malformations, steno-occlusive arterial disease, and subarachnoid hemorrhage [16–18, 24]. Studies also demonstrated the feasibility of q-DSA for the evaluation of peri-therapeutic hemodynamic changes for carotid stenting [25]. Keita et al demonstrated the predictive value of hemodynamic parameters derived from q-DSA in hyperperfusion phenomenon after carotid artery stenting and angioplasty [26]. Jayme et al confirmed that mean transit time generated from q-DSA could predict hemorrhagic transformation in patients following endovascular thrombectomy [14]. Multiple hemodynamic parameters can be obtained from q-DSA, including arrival time, peak density, AUC, TTP, full width half maximum, etc. Several factors that affect the quantitative analyses of DSA include the location of the catheter, injection protocol, and frame rate [27, 28]. However, the differences in TTP in different sites can reflect relative temporal delays, offsetting differences in catheter location and injection. Therefore, TTP is considered one of the most robust parameters in clinical practice, regardless of the DSA acquisition protocol [25, 29].

This study revealed that prolongation of preprocedural rTTP of the target vessel and shortened trans-stenotic TTP at the preprocedural DSA imaging were associated with recurrent events. Prolonged TTP reflects low blood flow velocity implying a low local blood pressure gradient and therefore decreased perfusion. In addition, lumen loss due to stenosis lesions increases focal flow velocity, which is manifested as a shortening of trans-stenotic TTP difference. The association between reduced blood flow and recurrent ischemic events had been suggested in patients with vertebrobasilar artery stenosis [30]. Insufficient perfusion, together with artery-to-artery embolism, and plaque extension over the orifice of penetrating arteries have been hypothesized as the main mechanism of ischemic events [1, 31].

Although PTAS procedures significantly improved perfusion status, patients with more restricted baseline blood flow still showed higher risks of stroke recurrence. Restricted blood flow often indicates more severe and continuous cerebral hypoperfusion. Chronic cerebrovascular hypoperfusion may injure the vascular endothelium, resulting in local neurovascular unit dysfunction and disruption [32–34]. The mechanisms result in permanent cerebral damage [33]. Therefore, even if the blood flow status is corrected, the residual risk of patients with worse baseline blood flow status is still higher than that of other patients.

The decreased perfusion could be due to insufficient upstream pressure or high downstream resistance. Apart from the insufficient perfusion pressure that can be improved after PTAS procedures, prolonged TTP may also be the result of increased downstream resistance, which was caused by chronic damage due to high-risk cardiovascular factors [35]. Comorbidities worsen cerebral circulation through several mechanisms, including hypoperfusion, diminished autoregulatory capacity, and increased permeability of the blood–brain barrier [36]. This highlights the importance of secondary prevention to prevent recurrent ischemic stroke. For patients with poor baseline hemodynamic status, even underwent PTAS procedure, more attention and intensive medical treatment were still needed. In other words, high-risk patients need more close monitoring, intensified follow-ups, and individualized secondary prevention strategies, such as longer duration of the dual antiplatelet therapy and intensive statin therapy.

Despite interesting and useful findings, limitations exist. Firstly, due to the high image quality requirements, a considerable number of patients failed to meet the inclusion criteria, resulting in a possible selection bias. Secondly, the semi-quantitative assessment from 2D DSA images can only infer hemodynamics, and there is a lack of more pulsatile flow parameters to reveal the detailed hemodynamic state. Thirdly, the small sample size and low heterogeneity of the population may limit the generalizability of conclusions. Therefore, further prospective studies with large samples are warranted to validate these findings.

## Conclusions

In the current study, q-DSA was applied to evaluate the pre- and postprocedural hemodynamic status of ICAS patients receiving PTAS. Although PTAS procedures could significantly improve blood flow status, patients with more restricted blood flow at baseline still showed a higher risk of stroke recurrence. More precisely, posterior ICAS patients with the prolonged TTP of the target vessel and shortened trans-stenotic TTP difference at the preprocedural DSA imaging were at a significant higher risk of stroke recurrence in the long-term follow-up. Therefore, baseline hemodynamic information obtained from q-DSA could be used for risk stratification in patients with ICAS.

### Supplementary information

ESM 1 (PDF 261 kb)

## Abbreviations

DSA: Digital subtraction angiography
HDL: High-density lipoprotein
ICAS: Intracranial atherosclerotic stenosis
IQR: Interquartile range
NLR: Neutrophil-to-lymphocyte ratio
PTAS: Percutaneous transluminal angioplasty and stenting
q-DSA: Quantitative digital subtraction angiography
rTTP: Relative time to peak
SD: Standard deviation
TIA: Transient ischemic attack
VA: Vertebral artery
WASID: Warfarin-Aspirin Symptomatic Intracranial Disease

## Declarations

### Guarantor

The scientific guarantor of this publication is Xinfeng Liu, MD, PhD, Department of Neurology, Jinling Hospital, Medical School of Nanjing University, email: xfliu2@vip.163.com.

### Statistics and biometry

One of the authors has significant statistical expertise.

### Study subjects or cohorts overlap

The study subjects or cohorts have not been previously reported in elsewhere.

### Methodology

- Retrospective
- Observational
- Performed at one institution
