# Supplementary material for: Angiography-based hemodynamic features predict recurrent ischemic events after angioplasty and stenting of intracranial vertebrobasilar atherosclerotic stenosis
Source: Eur Radiol. 2023 Sep 19;34(4):2352–63. doi: 10.1007/s00330-023-10209-x (PMC10957605; doi:10.1007/s00330-023-10209-x)
Supplement: Supplementary file 1 — (PDF 261 kb) [file 330_2023_10209_MOESM1_ESM.pdf]

Angiography-based hemodynamic features predict recurrent ischemic events after angioplasty and stenting of intracranial vertebrobasilar atherosclerotic stenosis

Electronic Supplementary Material

Supplemental Figure 1: The flow chart of patient selection in this study.

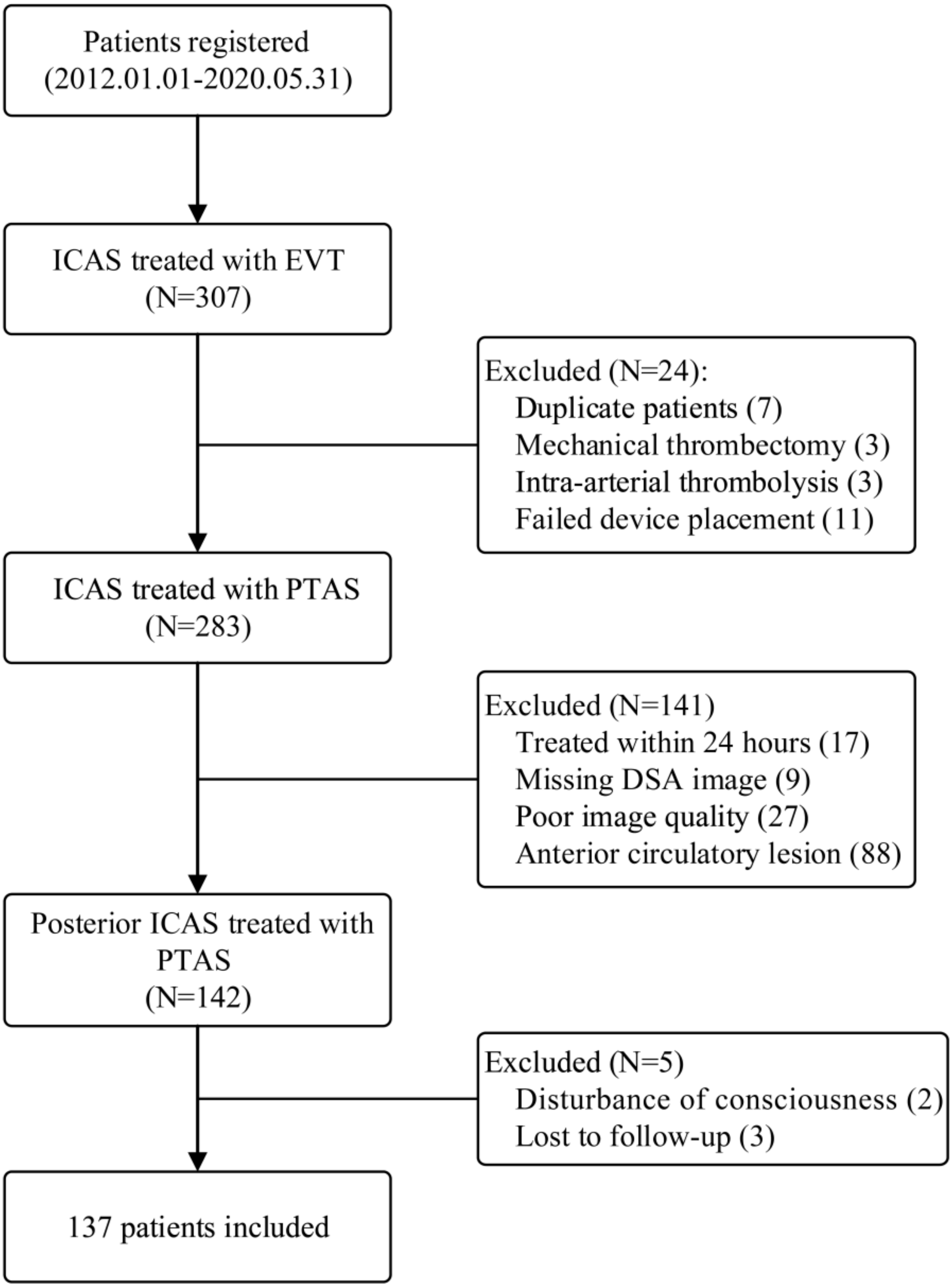

Abbreviations: EVT, endovascular therapy; PTAS, percutaneous transluminal angioplasty and stenting; DSA, digital subtraction angiography.
